# A Manual of Medical Jurisprudence and Toxicology

**Published:** 1893-09

**Authors:** 


*A Manual of Medical Jurisprudence and Toxicology.* By HENRY C. CHAPMAN, M.D. Pp. 237. Philadelphia: W. B. SAUNDERS. 1892.

This little work is essentially the course of lectures given by the author to his pupils at Jefferson Medical College, Philadelphia. It in no wise pretends to be a complete work on the subject, and only treats of such parts, which as coroner's physician in Philadelphia, Dr. Chapman regards as most important for practical purposes.

The law of coroners and of lunacy commented on, is of course that of America, and does not apply to this country. The few pages of Chapter xiii. are all that are given to the law of lunacy, certificates, &c., and the many intricate questions connected with the forms of the disease, the medico-legal aspects of insanity, criminal responsibility, &c. The chapter on toxicology omits any reference to several important poisons. The work is also so extremely condensed, that, used as notes for looking over before an examination, many of its chapters might be found useful by the student after the necessary preliminary reading of our own accredited authors.

There is one point worthy of note, and that is the excellence of the woodcuts, and especially of the diagrams, in the book, the latter being a good example, in their display and manner, of how such things ought to be done. The book, however, can hardly be expected to take the place with us of such a work as that of Dr. Aubrey Husband.

